# Stable transformation of an episomal protein-tagging shuttle vector in the piscine diplomonad *Spironucleus vortens*

**DOI:** 10.1186/1471-2180-8-71

**Published:** 2008-04-29

**Authors:** Scott C Dawson, Jonathan K Pham, Susan A House, Elizabeth E Slawson, Daniela Cronembold, W Zacheus Cande

**Affiliations:** 1 Department of Microbiology, 255 Briggs Hall, One Shields Ave., UC-Davis Davis, CA 95616, USA; 2 Molecular and Cell Biology, 345 Life Science Addition, UC-Berkeley, Berkeley, CA 94720-3200, USA

## Abstract

**Background:**

Diplomonads are common free-living inhabitants of anoxic aquatic environments and are also found as intestinal commensals or parasites of a wide variety of animals. *Spironucleus vortens *is a putatively commensal diplomonad of angelfish that grows to high cell densities in axenic culture. Genomic sequencing of *S. vortens *is in progress, yet little information is available regarding molecular and cellular aspects of *S. vortens *biology beyond descriptive ultrastructural studies. To facilitate the development of *S. vortens *as an additional diplomonad experimental model, we have constructed and stably transformed an episomal plasmid containing an enhanced green fluorescent protein (GFP) tag, an AU1 epitope tag, and a tandem affinity purification (TAP) tag. This construct also contains selectable antibiotic resistance markers for both *S. vortens *and *E. coli*.

**Results:**

Stable transformants of *S. vortens *grew relatively rapidly (within 7 days) after electroporation and were maintained under puromycin selection for over 6 months. We expressed the enhanced GFP variant, eGFP, under transcriptional control of the *S. vortens *histone H3 promoter, and visually confirmed diffuse GFP expression in over 50% of transformants. Next, we generated a histone H3::GFP fusion using the *S. vortens *conventional histone H3 gene and its native promoter. This construct was also highly expressed in the majority of *S. vortens *transformants, in which the H3::GFP fusion localized to the chromatin in both nuclei. Finally, we used fluorescence *in situ *hybridization (FISH) of the episomal plasmid to show that the transformed plasmid localized to only one nucleus/cell and was present at roughly 10–20 copies per nucleus. Because *S. vortens *grows to high densities in laboratory culture, it is a feasible diplomonad from which to purify native protein complexes. Thus, we also included a TAP tag in the plasmid constructs to permit future tagging and subsequent purification of protein complexes by affinity chromatography via a two-step purification procedure.

**Conclusion:**

Currently, progress in protistan functional and comparative genomics is hampered by the lack of free-living or commensal protists in axenic culture, as well as a lack of molecular genetic tools with which to study protein function in these organisms. This stable transformation protocol combined with the forthcoming genome sequence allows *Spironucleus vortens *to serve as a new experimental model for cell biological studies and for comparatively assessing protein functions in related diplomonads such as the human intestinal parasite, *Giardia intestinalis*.

## Background

Diplomonads are common microaerophilic protists in anoxic environments, and most known diplomonads have been isolated as either commensals or parasites of the metazoan intestinal tract [1]. Nearly a score of free-living or commensal diplomonads have been described and classified phylogenetically [1,2]. Binucleate diplomonads are believed to be related to mono-nucleate anaerobic protists such as retortamonads and enteromonads [1,3,4]. *Spironucleus vortens *is a putatively commensal diplomonad originally isolated from the intestinal lumen of the freshwater angelfish *Pterophyllum scalare*. *S. vortens *is typically described as a pear-shaped microaerophile, measuring 12.5 – 20.5 μm in length and 5.0 – 11.2 μm in width [5]. Like all described diplomonads, *S. vortens *has two nuclei and eight flagella. One pair of axonemes is enclosed by a flagellar pocket, termed a "cytostomal canal", and each of the recurrent flagella lie in a separate flagellar pocket [5,6]. As widespread human parasites, members of the genus *Giardia *(e.g. *Giardia intestinalis*) are perhaps the most well known of the diplomonads and are unique among diplomonads due to the presence of the ventral disc, which is a novel microtubule organelle that facilitates attachment to the intestinal microvilli in vertebrate hosts [7,8]. *S. vortens *lacks the ventral disc structure.

In the context of host-microbe interactions, microbes are traditionally described as free-living, commensals, symbionts, or parasites/pathogens [9]. These historical descriptions, however, do not incorporate the contemporary understanding that there exists a continuum of symbiosis in nature ranging from truly free-living to obligately parasitic (reviewed recently in [9,10]). Based on currently available data, *S. vortens *likely lies midway along that continuum as either a commensal or an opportunistic parasite. For instance, *S. vortens *has been found in the intestines of healthy angelfish [11], does not always cause host damage following infection [11], and does not cause systemic infection independent of special conditions [5]. Furthermore, bacteria have also been found in "hole-in-the-head" disease lesions in fish [12]. Thus it is conceivable that *S. vortens *may be indirectly accumulating in lesions due to this bacterial presence rather than directly causing the lesions. Lastly, other commensal diplomonads such as *Spironucleus torosa *attach to the intestinal mucosa without causing detectable host damage [2]. Thus in spite of its initial classification as a parasitic pathogen of angelfish, a more conservative conclusion is that *S. vortens *is either a commensal or, under specific conditions, an opportunistic parasite [11]. Further investigation is required to determine if *S. vortens *is a non-pathogenic commensal (causing little or no damage to the host), an opportunistic parasite (causing damage to the host under special conditions), or an obligate parasite (always causing damage to the host) [13].

To date, all molecular genetic studies of protein function in diplomonads have employed the common intestinal parasite, *Giardia intestinalis *(reviewed in [14]). Consequently, there is little information available regarding molecular and cellular aspects of general diplomonad biology beyond computational inferences of function [15] and/or ultrastructure in diverse diplomonads [2,5,11,16-19]. We describe here a method for the transformation of recombinant constructs in *S. vortens*, thus providing an essential tool to further investigate *Spironucleus' *potential for parasitism in angelfish as well as for comparative cell biological, functional genomic, and evolutionary studies in other diplomonads. Specifically, we designed an episomal shuttle vector to contain an enhanced green fluorescent protein (eGFP) tag, an AU1 epitope tag, and a tandem affinity purification (TAP) tag. Both the eGFP and AU1 epitope tags permit visualization of protein fusions both in live and fixed cells, respectively. Fusion of *S. vortens *proteins with the TAP tag would permit the purification and identification of interacting proteins by standard proteomic approaches [20].

## Results

**Figure 1:**
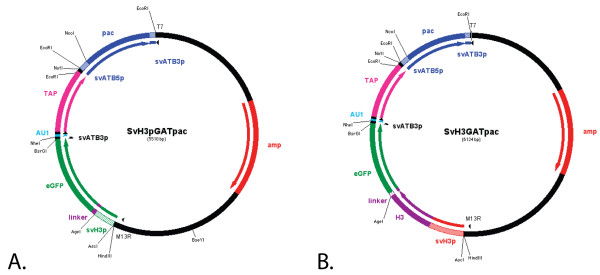
**Schematic vector maps of SvH3P.pac and SvH3G.pac**. A) Schematic plasmid map of the SvH3P.pac shuttle vector that includes an *S. vortens *H3 promoter for transcription of eGFP (enhanced GFP), an AU1 epitope tag, and a tandem affinity purification (TAP) tag for affinity purification (GAT = GFP+AU1+TAP). This plasmid also includes a puromycin resistance gene (pac) for selection in *S. vortens *flanked by the α-tubulin 5'UTR and 3'UTR regions (SvATB5p and SvATB3p), and an ampicillin resistance gene (amp) for antibiotic selection in *E. coli*. B) Schematic plasmid map of the SvH3G.pac shuttle vector. In this plasmid, the *S. vortens *histone H3 gene (including ~100 bp of the H3 histone native promoter (SvH3p) was fused to and includes a 12 amino acid hydrophobic linker, as well as other selectable markers (see Figure 1A). Relevant restriction sites are also highlighted in the vector maps, as well as M13R and T7 sequencing primer regions.

As a first step in developing a molecular toolkit that can be used to study protein function, we constructed, stably transformed, and maintained episomal shuttle vectors (Figure 1A and 1B) in *S. vortens *by adapting a methodology used in *Giardia *to generate stable transformants [21]. These constructs also contained selectable antibiotic resistance markers for both *S. vortens *(puromycin resistance) and *E. coli *(ampicillin resistance). We demonstrate here a highly stable transformation of these constructs in *S. vortens*, and the ability to express GFP fusion constructs and visualize them in live and fixed cells in two stable GFP-expressing strains: SvH3P, which expresses GFP under the control of a *S. vortens *histone H3 promoter, and SvH3G, which expresses a histone H3::GFP fusion under the control of the native promoter.

### Stable transformation confirmed by RT-PCR and FISH of the episomal plasmids

**Figure 2:**
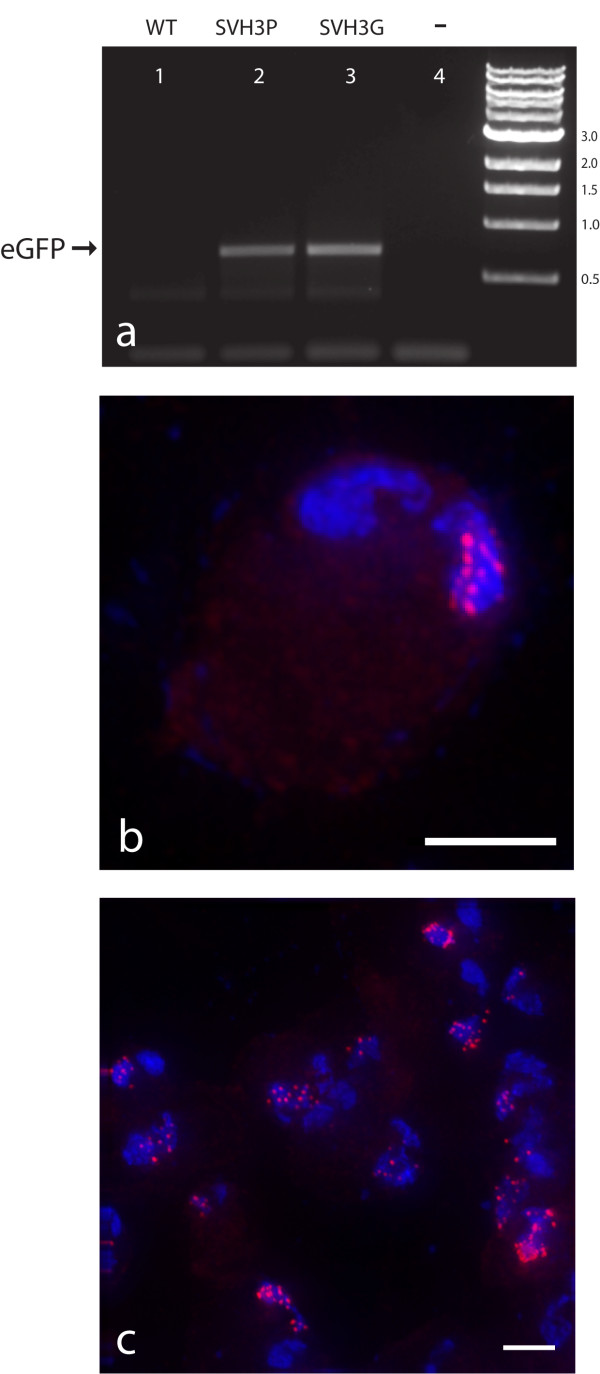
**Expression of GFP in the SvH3P and SvH3G transformed strains**. Shown in panel A): expression of GFP mRNA as measured by RT-PCR. Lane 1) untransformed wild-type *S. vortens*; Lane 2) transformed SvH3P strain (full-length GFP with the SvH3 promoter); Lane 3) transformed SvH3G strain (H3::GFP fusion with the native H3 promoter); Lane 4) negative control (no template RNA added). The expected size of the eGFP amplicon (721 bp) is indicated by the arrow. To determine the copy and locations of the transformed plasmids, we used fluorescence *in situ *hybridization of a Cy3-labelled probe to the SvH3P.pac episomal plasmid in the transformed SvH3P strain. Panel B) shows a single *S. vortens *cell exhibiting multiple fluorescent foci (10–15) in the right nucleus. Panel C) shows a microscopic field of several *S. vortens *cells indicating the range of foci/nucleus (~10–20). Note that plasmids only localize to one nucleus (either left or right). Cy3-labelled FISH probe = red, DAPI = blue. Scale bars = 2 μm.

Using our protocol for transformation of *S. vortens*, we created three stable cell lines: SvPAC (expressed puromycin resistance (pac) gene); SvH3P (GFP expressed by the H3 histone promoter); and SvH3G (a histone H3::GFP fusion) (see Methods). The SvH3P and SvH3G strains were created by transfecting SvH3P.pac (Figure 1A) and SvH3G.pac (Figure 1B), respectively, into wild type *S. vortens *cell cultures. To verify that the putatively transformed strains expressed GFP, we performed RT-PCR of the GFP gene in both the SvH3P and SvH3G transformed strains (Figure 2a). We observed strong amplification of a band corresponding to the correct size of the GFP using RNA from both the SvH3P and SvH3G transformed strains as templates. No GFP expression was observed in untransformed cells or in negative (no RNA template added) controls (Figure 2a).

The number of plasmids that were transformed per nucleus per cell was estimated using fluorescence *in situ *hybridization (FISH) with a DNA probe against a 5 Kb amplified region of the episomal plasmid used in the transformed SvH3P strain. As seen in Figure 2b, we estimate that roughly 10–20 plasmids were present per transformed nucleus. Importantly, for all cells transformed with the SvH3P.pac construct, the plasmid FISH probes localized to only one nucleus (roughly 250 total cells were observed) (Figure 2c). We were also able to recover the plasmids from the transformed SvH3P and SvH3G strains and transform them back into *E. coli *(data not shown) at high efficiency.

### GFP expression visualized using epifluorescent microscopy

**Figure 3:**
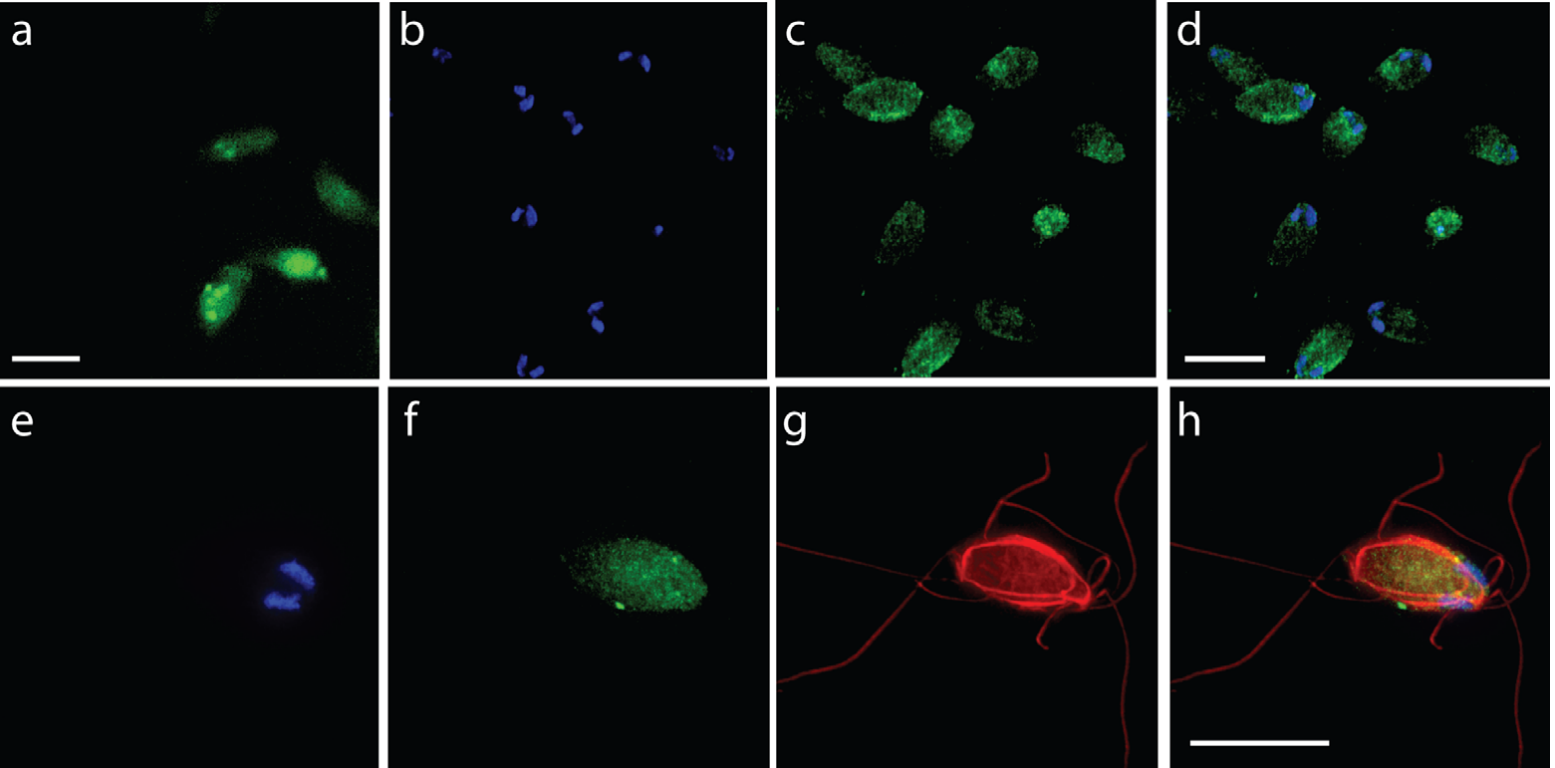
**GFP localization in the transformed SvH3P strain**. Interphase cytoplasmic GFP localization in live and fixed cells, with immunolocalization of the microtubule cytoskeleton in *S. vortens *trophozoites (DAPI, blue; GFP, green; anti-α-tubulin, red). UPPER PANELS: Panel (a) shows the GFP fluorescence of a field of live *S. vortens *cells. Panels (b-d) show a different field of fixed *S. vortens *cells, with (b) marking the DAPI-stained nuclei of the cells, (c) marking the cytoplasmic GFP localization of the cells, and (d) showing the merged image of panels (b) and (c). The upper panels show that the GFP expressed from the *S. vortens *H3 promoter had a cytoplasmic localization (with some foci) in both live cells (a) and fixed cells (c-d). Cytoplasmic localization is particularly obvious in fixed cells with respect to the two nuclei (see DAPI-stained nuclei in (b)). The upper panels also illustrate a high number of cells (>75%) expressing GFP, as is shown in the field of fixed cells (b-d). LOWER PANELS: Panels (e-h) show the same fixed *S. vortens *cell, with (e) marking the DAPI-stained nuclei, (f) marking the GFP localization, (g) marking the microtubule cytoskeleton, and (h) showing a merged image of panels (e-g). In the lower panels, cytoplasmic GFP staining is shown (f, h) in striking contrast with DAPI-labeled chromatin (e) and anti-α-tubulin immunostaining (g), which defines the eight flagella and the lateral ridges of the microtubule cytoskeleton (red). Images are representative of GFP localizations observed in over 250 individual cells. Scale bar = 2 μm.

**Figure 4:**
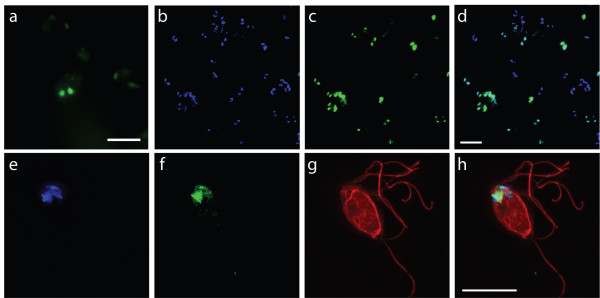
**H3::GFP localized to both nuclei in the transformed SvH3G strain**. Nuclear localization of H3::GFP and comparative immunolocalization of the microtubule cytoskeleton in *S. vortens *trophozoites (DAPI, blue; H3::GFP, green; anti-α-tubulin, red). UPPER PANELS: Panel (a) shows the GFP fluorescence of a field of live *S. vortens *cells. Panels (b-d) show a different field of fixed *S. vortens *cells, with (b) marking the DAPI-stained nuclei of the cells, (c) marking the nuclear GFP localization of the cells, and (d) showing the merged image of panels (b) and (c). The upper panels further illustrate that the H3::GFP transgene is expressed in a high proportion of cells (b-d). LOWER PANELS: Panels (e-h) show the same fixed *S. vortens *cell, with (e) marking the DAPI-stained nuclei, (f) marking the GFP localization, (g) marking the microtubule cytoskeleton, and (h) showing a merged image of panels (e-g). In the lower panels, staining of a single cell with anti-α-tubulin immunostaining (g, h in red) labels the eight axonemes and the lateral ridges of the microtubule cytoskeleton. The H3::GFP expressed from the native promoter localizes to both nuclei in both live cells (a) and fixed cells (b-h), and the H3::GFP transgene co-localizes to the DAPI-stained nuclei (b-f), but not to the microtubule cytoskeleton as visualized by anti-α-tubulin immunostaining (g, h). Though H3::GFP co-localizes with DAPI, H3::GFP has a more punctate staining pattern. Images are representative of GFP localizations observed in over 250 cells per strain. Scale bar = 2 μm.

In both the SvH3P and SvH3G strains, we observed significant GFP expression in live, highly motile *S. vortens *cells (see Figures 3, 4, and see Additional files 1 and 2). To determine the subcellular localization of the GFP fusion proteins and to qualitatively assess the efficiency of our transformation protocol, both the SvH3P and SvH3G strains were fixed with 1% paraformaldehyde (a procedure that is compatible with the maintenance of GFP fluorescence after fixation) and microtubules were immunostained with anti-α-tubulin antibody (Figure 3g–h, Figure 4g–h) using a protocol previously described for *Giardia intestinalis *[22]. Images were acquired in a three-dimensional stack using deconvolution microscopy, and 2D projections of 3D stacks were created as described (Methods). For each transformed strain, we imaged 250 cells and visually quantified the number of GFP-expressing cells. As shown in Figure 3, GFP fluorescence was observed throughout the cytoplasm of the SvH3P strain, which used a *S. vortens *histone H3 promoter (identified from the in-progress genome project) to drive expression of GFP. In Figure 3b–d, a high percentage of cells (~78%) express GFP at easily detectable levels as seen by either epifluorescent microscopy using direct cell counts (N = 250) or as seen in live videomicroscopy (Figure 3a).

We also localized GFP expression in the SvH3G strain (*S. vortens *histone H3 gene fused in frame to a C-terminal eGFP tag) and imaged the GFP localization in anti-α-tubulin immunostained cells (as above). As seen in Figure 4 and Figure S2, the GFP fluorescence in the SvH3G strain was exclusively localized to both nuclei, as expected for the histone localization to euchromatin. Based on the imaging of 250 cells, we visualized detectable levels of GFP expression in about 66% of the transformed cells (Figure 4b–d).

Additionally, anti-α-tubulin immunostaining of the microtubule cytoskeleton of *S. vortens *(Figure 3g–h, Figure 4g–h) shows in detail the microtubules of the lateral ridges, the microtubules surrounding the flagellar pockets, and the microtubules of the counter-crossing ridges [8,23].

## Discussion

The majority of proposed eukaryotic lineages are represented by microbial eukaryotes [24-30]. Most of our knowledge of eukaryotic biological processes derives, however, from studies of more recently evolved (and phylogenetically restricted) macroscopic eukaryotic model organisms such as fungi and metazoans. Furthermore, progress in protistan functional and comparative genomics is generally complicated by the fact that most free-living protists have not been maintained in pure culture, and only a handful have been developed as molecular experimental systems. In the absence of genetic manipulation, classical studies in protistan biology have generally had to rely solely upon descriptive ultrastructural or comparative genomic studies.

In spite of this, current in-progress and completed genome projects of diverse microbial eukaryotes are transforming our understanding of eukaryotic biology [31]. Emerging genomic and EST data from axenically cultivated diplomonads such as *S. vortens *[32], *S. salmonicida *[15], and *G. intestinalis *[33] highlights the importance of developing functional genomic methodologies to complement prior descriptive ultrastructural or comparative genomic studies. Stable transformation of recombinant genes, such as that which is presented here for *Spironucleus vortens*, is critical toward the development of modern reverse genetic and proteomic strategies that permit a functional understanding of diplomonad biology on par with other experimental systems.

### Stable transformation and maintenance of an episomal shuttle vector

Electroporation takes advantage of the fact that membranes in cells essentially act as electrical capacitors [34]. Transformation of eukaryotic microbes via electroporation is generally applicable and reproducible [35-41]. We have modeled our *Spironucleus vortens *transformation protocol after the *Giardia intestinalis *transformation protocol developed by Singer et al. [21]. Based on both microscopic and biochemical analyses of transformants (Figures 2, 3, 4, and see Additional files 1 and 2), the use of this protocol results in stable transformants of *S. vortens *with a minimal efficiency of 50%. Additionally, we have demonstrated that the *S. vortens *H3 histone promoter is sufficient to drive transcription of GFP (Figure 2a, Figure 3 and see Additional file 1), and that a histone H3::GFP fusion protein is expressed (Figure 2a) and localizes to chromatin in both nuclei (Figure 4 and see Additional file 2). We were able to successfully passage all transformed *S. vortens *strains (SvPAC, SvH3P, and SvH3G) under puromycin selection for a period of over six months, indicative of stable rather than transient transformation.

Finally, we confirmed that the transformed episomal plasmid localized to only one nucleus per cell (Figure 2b–c), as is the case in binucleate *Giardia *[22,42,43]. For neither organism do we understand why only one nucleus is transformed, but this result is consistent with the idea that transformation is a rare stochastic event that only affects one of the two nuclei. However, the presence of multiple episomal plasmids (~10–15) in only one nucleus/cell (Figure 2b) suggests that *S. vortens *undergoes mitosis in a manner similar to that of *Giardia intestinalis *[22]. In *Giardia*, the two nuclei do not fuse during mitosis but instead behave autonomously so that only one copy of each parental nucleus is inherited by each daughter [22]. In *Giardia*, both nuclei migrate to the line of bilateral symmetry and are stacked one above the other (one ventral and one dorsal). During anaphase, the nuclei divide and separate such that each daughter cell obtains a nucleus from each plane (one ventral nucleus and one dorsal nucleus) [22]. This inferred mitotic mechanism explains the segregation of episomal plasmids to one nucleus in *S. vortens *and supports the theory that the two nuclei of *S. vortens *do not fuse during mitosis. This state of singly transformed nuclei would be perpetuated throughout the population, although there is no information regarding whether both nuclei in *S. vortens *are transcriptionally active or whether they contain the same genetic material.

### Potential for proteomic analyses in S. vortens

The *Spironucleus *genome project was begun under the Community Sequence Program (CSP) through a collaboration with the DOE-Joint Genome Institute (JGI) in 2004. Currently, over 15,000 ESTs have been sequenced, in conjunction with roughly 4× genome coverage of the 16 Mb genome (unpublished data). We have engineered the SvGAT.pac shuttle vector so that a GFP fusion construct can easily be converted into one with an AU1 fusion tag by a double restriction digest with AgeI/NheI; alternatively, a tandem affinity purification (TAP)-tagged fusion protein can be created using an AgeI/AvrII restriction digest. When religated, the initial GFP gene fusion will remain in frame with either the AU1 epitope tag or TAP tag.

*S. vortens *grows robustly in axenic culture in the laboratory to densities of roughly 1 × 10^7 ^cells/ml, and tolerates temperatures ranging from 5–34°C [6]. For the experiments detailed in this paper, we have observed rapid growth of *S. vortens *at room temperature (25°C). Hence, due to its rapid growth in laboratory culture and its nearly completed genome sequence, *Spironucleus *seems a feasible diplomonad from which to purify proteins and protein complexes. For this reason, we included a TAP tag in the plasmid constructs to permit the tagging of proteins and subsequent purification of protein complexes by affinity chromatography via a two-step purification procedure. This proteomic strategy for identifying interacting proteins has been widely used in other organisms [20,44]. The affinity-purified complexes can then be eluted with EGTA and analyzed by SDS-PAGE and MALDI-TOF, and/or directly subjected to proteolysis and analysis by liquid chromatography-coupled mass spectrometric analysis (LC-MS-MS) [20,44]. Future proteomic-based work with *S. vortens *could include the purification of protein complexes followed by mass spectrometry to analyze protein components.

### Using protein-tagging approaches to determine the putative parasitism of S. vortens

The use of this stable transformation protocol to generate fluorescently tagged *S. vortens *strains could help confirm whether or not *S. vortens *is actually an obligate parasite in angelfish. Briefly, fluorescently tagged *S. vortens *could be used to confirm that: 1) *S. vortens *is present in all cases of "hole-in-the-head"-diseased angelfish; 2) inoculation of healthy angelfish with tagged *S. vortens *results in disease; and, 3) isolated, tagged *S. vortens *strains from newly diseased angelfish are also infectious. Following inoculation of these transformed *S. vortens *cells back into healthy angelfish, resultant "hole-in-the-head" lesions (if any) could be imaged and/or quantified using GFP fluorescence, which (if found) would indicate that *S. vortens *plays and obligately pathogenic role in angelfish. At this time, no such experimental verifications of the obligate parasitism of *S. vortens *have been performed.

## Conclusion

With a nearly completed genome sequence and a protocol for stable transformation, *Spironucleus vortens *represents a new experimental system with which one can study comparative cell biology in diplomonads. Because it is putatively commensal and lacks the ventral disc present in *Giardia *[8,11], the study of the cytoskeleton in *S. vortens *could provide insight into *Giardia's *adaptations to parasitism – including the evolution of the ventral disc. Functional genomics in *S. vortens *also offers a strategy to test the hypothesis of its obligately parasitic role in angelfish. Finally, ongoing comparative and functional genomic analyses of putative commensal diplomonads (i.e. *Spironucleus vortens *[32]) as well as obligately parasitic diplomonads (i.e., *Giardia intestinalis *[33]) are critical toward validating hypotheses regarding the basal phylogenetic position and genomic minimalism of the diplomonads [45].

## Methods

### Culture conditions

*Spironucleus vortens *(ATCC 50386) was cultivated in 13 ml polypropylene screw cap tubes (Falcon) in 12 ml of medium at 25°C using previously described culture methodologies [5]. Cultures were diluted ten-fold every 3–5 days for over six months. Before selection in medium with 50 μg/ml puromycin, transformed *S. vortens *strains were grown in 24-well plates sealed in BioBags (Fisher) to maintain a low-oxygen tension.

### Construction of GFP/AU1/TAP vector

To create a stable episomal plasmid vector for transformation of *S. vortens*, we modified the previously constructed pMCS-GFP vector from *Giardia intestinalis *[46] to include *S. vortens *promoter regions for the puromycin resistance (pac) gene and for the GFP-AU1-TAP tag (Figure 1). Specifically, we modified the puromycin resistance (pac) selectable marker to include the *S. vortens *α-tubulin (atb) promoter region (roughly 100 nucleotides upstream of the *S. vortens *α-tubulin (atb1) gene) and the α-tubulin 3'UTR (roughly 50 nucleotides downstream).

We added the *S. vortens *α-tubulin promoter by PCR-amplifying the pac gene from the pMCS-GFP vector using the following oligonucleotide primers that included the *S. vortens *atb1 promoter region and 3'UTR: atbpacF: **5'GAA TTC ***TTA CGG TAA AAA TAA GAC CAG CGT CCG AAA TTT TGG CCA AAA ATT TTC CGG AAT TTT CGT ACC ATC TAT TCA TCC *ATG GGC ACC GAG TAC AAG-3' and 3atbpacR: 5'**GAA TTC ***GCT ACT TAA AAT ATA TTG AAA CTT ACT TAA AAT ATT GAA AAT AAT AAA CAG AAA GAT CAC T*CG AGG GCA CCG GGC TTG CGG G3' (bold= EcoR1 site, italics = promoter regions). This PCR product was first subcloned into the pCR2.1 TOPO-TA vector (Invitrogen), digested with EcoRI, and ligated into the pMCS-GFP vector to create the SV.pac construct. Next, we replaced the GFP of the SV.pac vector with a triple protein tag (GFP-AU1-TAP tag, or "GAT") driven by the *S. vortens *H3 histone promoter as identified from the in-progress *S. vortens *Genome Project (see Availability and requirements section for URL). These three protein tags each have a stop codon and short putative polyA signal region (TTTCTTT). The TAP tag and AU1 tagged portions of the GAT tag were amplified using PCR with the following oligonucleotide primers SVGATF: 5' **TGT ACA **AGT GAT TTC TTT GTT TAT TAT GCT AGC GAC ACG TAC CGA TAC ATA*TGA TTT CTT TGT *TTA TTA TCC TAG GAT GGA AAA GAG AAG ATG G-3' and SVGATR: 5'-GCG GCC GCA TAA *TAA ACA AAG AAA *TCA GGT TGA CTT CCC CGC G**GA ATT C**G 3' using the pKG1810 plasmid as a template [47]. The oligonucleotide PCR primers included an AU1 epitope tag (DTYRYI, codons underlined) and a putative polyA signal sequence (italics) and BsrGI/EcoRI restriction sites (bold). The GAT insert was initially cloned into the TOPO-TA vector (Invitrogen) and recombinant plasmids were digested with BsrGI and EcoRI. The GAT insert was gel-purified and ligated into the BsrGI/EcoRI sites downstream of the GFP sequence in the SV.pac vector to create *S. vortens *shuttle vector SvGAT.pac.

To drive transcription of GFP fusion proteins, we PCR-amplified an *S. vortens *H3 histone promoter (H3P) from *S. vortens *genomic DNA using the oligonucleotide PCR primers: svh3PF: 5'GGC GCG CCG GAA ACG AAC TTT CGG AGT ATG CCG CTC GG-3' and svh3PR: 5'-ACC GGT AGC ATC TGC TTG ATT TCT GAA AGG GGA AGG G3'. This PCR product was initially cloned into the TOPO-TA vector (Invitrogen), and the insert was recovered by digestion with AscI/AgeI, then ligated into an AscI/AgeI digest of the SvGAT.pac vector. This created the SvH3P.pac vector that was subsequently used to transform *S. vortens *(see Figure 3).

Finally, the full-length histone H3 gene including its native promoter was ligated into the SvGAT.pac vector to create a H3::GFP C-terminal fusion in the construct SvH3G.pac. The *S. vortens *H3 histone gene and ~80 bp of its native promoter were amplified from *S. vortens *genomic DNA using the oligonucleotide primers svh3GF 5' **GGC GCG CC**T AAT CAG TAT AGG CGT CCC GGA TGT CCC GC-3' and svh3GR 5'-**ACC GGT **AGC TGG GCG GGG TTC ATC GCG TGG ACC CAG AC3' that included AscI and AgeI restriction sites (bold). This PCR amplicon was initially cloned into the TOPO-TA vector (Invitrogen), and the insert was recovered through an AscI/AgeI digest. The H3 histone was then ligated into the AscI/AgeI sites of the SvGAT.pac vector. This yielded the SvH3G.pac vector that was subsequently used to transform *S. vortens *(see Figure 4). The full-length sequences of the *S. vortens *α-tubulin and histone H3 genes are deposited in GenBank with accession numbers: EV099373 and DQ677672.

### Stable transformation of an episomal plasmid using electroporation

To determine the optimal concentration for puromycin selection, puromycin was added at varying concentrations (1 μg/ml, 5 μg/ml, 25 μg/ml, 50 μg/ml, and 100 μg/ml) to *S. vortens *cultures, and cell viability was assessed at 24–120 hour time points by trypan blue exclusion and manual counting of cells by hemacytometer [48]. At 50 μg/ml, puromycin selection was observed to have a maximal effect on *S. vortens *viability after 3 to 4 days.

For the electroporation of *S. vortens*, approximately 1 × 10^7 ^cells (one confluent 13 ml culture tube) was first centrifuged at 1500 × g for five minutes at 4°C. The pellet was then washed once in fresh medium, centrifuged again, and resuspended in 1 ml medium (approximately 10^7 ^cells/ml). Roughly 50 μg of the SvH3P.pac or the SvH3G.pac episomal plasmids were mixed gently with 300 μl of *S. vortens *cells in a 4 mm electroporation cuvette (BioRad) and incubated at 4°C for ten minutes. Various electroporation voltages (ranging from 325V to 425V) were used for initial studies, but the optimal transformation was achieved with GenePulserXL (BioRad) using the following conditions: 400V, 1000 μF, and 700 ohms.

Following electroporation, cuvettes were incubated on ice for 10 minutes, and then electroporated *S. vortens *were transferred into 13 ml polypropylene tubes with 12 ml of medium. The electroporated *S. vortens *cells were grown for 24 hours at room temperature without antibiotic selection. Electroporated cells were then diluted 1:10, 1:100, or 1:1000 in 1.5 ml of *S. vortens *medium with a final concentration of 10 μg/ml puromycin (CalBioChem) in a 24-well plate (ThermoFisher). The plates were incubated at room temperature in BioBags (ThermoFisher) to maintain a low oxygen tension required for optimal growth. After 4–9 days, putative transformants were transferred to 13 ml polypropylene tubes with fresh medium, and antibiotic selection was increased to a final concentration of 50 μg/ml puromycin. Strains were maintained for greater than six months under selection with 50 μg/ml puromycin, and were aliquoted to medium containing 9% DMSO for storage in liquid nitrogen.

### RT-PCR of GFP expression in stably transformed strains

*In vivo *expression of GFP fusions in the transformed *S. vortens *strains (SvH3P and SvH3G) was confirmed by RT-PCR. Following total RNA extraction of a 13 ml culture (~1.0 × 10^7 ^cells) using RNA-STAT (Tel-Test), RT-PCR was performed with ~300 ng of *S. vortens *RNA from the transformed strains using the Superscript™ One-Step RT-PCR with Platinum Taq Kit (Invitrogen) according the manufacturer's instructions, using two GFP-specific PCR primers: GFPF: 5' TGAGCAAGGGCGAGGACGTGTTCACGG 3' and GFPR 5' ATCACTTGTACAGCTCGTCCATGCCG 3'. The expected size of the GFP fragment amplified using these primers is 721 bp.

### Localization of the episomal plasmid SvH3P.pac to a single nucleus by fluorescence in situ hybridization

Based on prior work in *G. intestinalis *that showed localization of episomal plasmids to one nucleus (even during mitosis) [22,42,43], we were interested in determining the nuclear localization of episomal plasmids in *S. vortens*. Toward this end, we constructed a fluorescence *in situ *hybridization (FISH) probe to an AscI/AgeI fragment of the SvH3G.pac plasmid that lacked the histone H3 gene, using the incorporation of Cy3-labelled dUTPs by nick translation (Roche). The Cy3-labelled hybridization probe was precipitated in LiCl and used in hybridization experiments in the *S. vortens *SvH3P.pac strain as previously described (Yu. et al, 2001).

In brief, transformed cells were centrifuged at 1500 × g for 5 minutes, and the cell pellet was resuspended in one ml HBS, and fixed in a final concentration of 4% paraformaldehyde. *S. vortens *cell suspensions were then placed on poly-L-lysine-treated coverslips (0.1%) and allowed to attach for 30 minutes. Following fixation, coverslips were dehydrated in 70% ethanol, and rehydrated before use in 2× SSC. Coverslips were then treated with DNAse-free RNAse for 3 hours at 37°C, followed by permeabilization with 0.5% Triton X-100 at room temperature for fifteen minutes, and finally redehydrated in 70% ethanol for five minutes and 100% ethanol for five minutes. Prior to hybridization, slides were denatured for two minutes in 70% formamide/2× SSC at 70°C and dehydrated for five minutes each in cold 70% ethanol (-20°C) and cold 100% ethanol. Probes resuspended in 100% formamide were denatured at 95°C for 10 minutes and then kept on ice. Denatured probe (20 μl) was mixed with 10 μg each of salmon sperm DNA, and *Saccharomyces cerevisiae *tRNA, air dried, and finally resuspended in 10 μl of 100% formamide. For hybridizations, an equal volume of hybridization buffer (4× SSC, 20% dextran sulfate, and 4 mg/ml BSA) was to the denatured probe. Coverslips were placed face down on the hybridization/probe solution and covered with Parafilm. After overnight incubation at 37°C, the coverslips were washed in 2× SSC with 50% formamide at 37°C for 30 minutes, followed by 2× SSC at 37°C and 1× SSC at room temperature for 30 minutes each and then placed on slides with ProLong anti-fade mounting medium. Three-dimensional images of *in situ *hybridizations were collected using epifluorescence deconvolution microscopy and processed as described below.

### Immunolocalization of the microtubule cytoskeleton with GFP fusion proteins

Transformed *S. vortens *strains in 12 ml culture tubes were fixed with 1% paraformaldehyde to maintain native GFP fluorescence, then centrifuged at 900 × g at 4°C. Pellets were washed twice in PEM buffer (100 mM PIPES, 1 mM EGTA, 0.1 mM MgSO_4_) and attached to poly-L-lysine-coated coverslips. *S. vortens *cells were permeabilized with 1 ml of 0.1% Triton X-100 for 10 minutes, and then washed three times in PEM. Coverslips were then incubated in PEMBALG (100 mM PIPES, 1 mM EGTA, 0.1 mM MgSO_4_,100 mM lysine, and 0.5% cold water fish skin gelatin (Sigma)) to block non-specific binding. Microtubules were counterstained by incubating coverslips with the monoclonal α-tubulin antibody TAT1 [49] diluted 1:75 in PEMBALG, and incubated at room temperature overnight. The TAT1 antibody was directly labeled (1:1) with a Zenon fragment conjugated to an Alexa 555 fluorophore (Molecular Probes, Inc.) at room temperature for 5 minutes. Following incubation, coverslips were washed three times in PEMBALG, then three times in PEM before mounting on slides with ProLong AntiFade with DAPI (Molecular Probes, Eugene, OR).

### Fluorescence Deconvolution Microscopy of live and fixed S. vortens

Three dimensional images were collected using SoftWorX image acquisition software (Applied Precision Inc, Issaquah, WA) on an Olympus IX70 wide-field inverted fluorescence microscope with an Olympus UPlanApo 100× (NA 1.35) or an Olympus UPlanApo 60× (NA 1.4) oil immersion objective and a Photometrics CCD CH350 camera cooled to -35°C (Roper Scientific, Inc, Tuscon, AZ). To visualize the cells in three dimensions, serial sections were acquired at 0.2 μm intervals, and data stacks were deconvolved using the SoftWorX deconvolution software. 2D projections were created from the 3D data sets using the DeltaVision image analysis software (Applied Precision Inc, Issaquah WA) for presentation purposes.

## List of Abbreviations Used

amp gene (ampicillin resistance gene); atb1 gene (α-tubulin gene); CBP (calmodulin binding protein); CSP (Community Sequence Program); DAPI (4',6-diamidino-2-phenylindole); eGFP (enhanced green fluorescent protein); FISH (fluorescence *in situ *hybridization); H3P (H3 histone promoter); LC-MS-MS (Liquid Chromatography/Mass Spectrometry/Mass Spectrometry); MALDI-TOF (Matrix Assisted Laser Desorption/Ionization – Time Of Flight); pac gene (puromycin resistance gene); RT-PCR (reverse transcription polymerase chain reaction); SV.pac (*S. vortens *vector containing pac gene driven by atb1 promoter); SvGAT.pac (*S. vortens *vector containing functional pac gene and GFP-AU1 TAP tag insert); SvH3G (*S. vortens *strain containing H3::GFP fusion driven by H3 promoter); SvH3G.pac (*S. vortens *vector containing functional pac gene and H3::GFP fusion driven by H3 promoter); SvH3P (*S. vortens *strain containing GFP expression driven by H3 promoter); SvH3P.pac (*S. vortens *vector containing functional pac gene and functional GFP gene driven by H3 promoter); SvPAC (*S. vortens *strain containing pac gene driven by atb1 promoter); TAP tag (tandem affinity purification tag); TEV (tobacco etch virus).

## Availability and requirements

*S. vortens *Genome Project: 

## Authors' contributions

SCD designed vectors and overall transformation strategy. SCD imaged immunostained transformed GFP strains and FISH experiments. SCD also managed experiments and wrote and revised the manuscript. JKP cloned and constructed *S. vortens *episomal plasmids. JKP conducted and analyzed transformation experiments, including RT-PCR analysis of GFP expression and reverse transformation of *E. coli*. JKP edited the manuscript. SAH performed live videomicroscopy of GFP-tagged strains and image analysis, and edited the manuscript. EES conducted FISH experiments to test localization of episomal plasmid, and edited the manuscript. DK tested sensitivity of *S. vortens *to various antibiotics and cloned the full-length α-tubulin gene and flanking regions before confirmation by genomic sequencing. DK also edited the manuscript. WZC managed experiments and edited manuscript.

## Supplementary Material

Additional file 1 **Live time-lapse epifluorescent microscopy movie of SvH3P strain. Live image analysis of the SvH3P strain using epifluorescent microcopy**. This short movie corresponds to the still image presented in Figure 3A, and demonstrates the cytoplasmic native GFP expression in the transformed *S. vortens *SVH3P strain. This movie demonstrates live GFP-tagged *S. vortens *and highlights the cytoplasmic localization of green fluorescent protein (GFP) expressed from the H3 histone promoter. This short movie corresponds to the still image presented in Figure 3A Click here for file

Additional file 2 **Live 3D movie of the SvH3G strain. Live image analysis of the SvH3G strain using epifluorescent microcopy**. This short three-dimensional stack presented as a movie corresponds to the still image presented in Figure 4A, and demonstrates the native GFP expression in both nuclei in the transformed SvH3G *S. vortens *strain. This movie shows the live histone H3 GFP-tagged *S. vortens *strain and highlights the nuclear localization of the H3G:GFP fusion. This short movie corresponds to the still image presented in Figure 4A Click here for file
